# Treatment of Carcinoma of the Uterus and Ovarian Disease without Operation

**Published:** 1897-11

**Authors:** 


### TREATMENT OF CARCINOMA OF THE UTERUS AND OVARIAN DISEASE WITHOUT OPERATION.

Dr. Robert Bell, of Glasgow, in an article in the *Scottish Med. and Surg. Journal*, states that in the past two years, in his own observation, a number of cases of epithelioma of the cervix have been completely cured by local treatment, supplemented by the administration of thyroid extract.

An abstract in the *New York Medical Journal*, July 17, 1897, gives these views of Dr. Bell quite fully. Dr. Bell refers to an interesting statement recently made by Snow, to the effect that hypodermic injection of morphine and cocaine delay, if they do not actually prevent, recurrence of scirrhus after operation. If this is a fact, he says, one is warranted in inferring that certain agents which are safe therapeutic remedies may be discovered to produce destructive effects upon morbid growths where these have established a habitat in a debilitated tissue. His experience during the past two years has fully confirmed this statement and inference, as a considerable number of instances have come under his observation where epithelioma of the cervix has completely disappeared under local treatment the object of which has been to promote a healthier condition of the organ, while this has been supplemented by the administration of thyroid extract. The local treatment consists in removing by the curette all the unhealthy tissue that can be reached, and the application afterward at frequent intervals of tampons saturated with a ten per cent. solution of ichthyol in glycerin.

He does not say that in every case such treatment is followed by a favorable result, yet in the majority of instances where the disease is in its initial stage and has not involved any other tissue than the cervix itself, one may calculate upon a most encouraging result. It appears that this method of treatment contains all the elements of success which can be alleged for complete hysterectomy. In a few instances where the disease has actually proceeded beyond the sphere of surgical interference complete recovery has followed this method of treatment. . . . . .

Extensive observations on this subject have taught him that the only form of cancer which takes its origin in the uterus is epithelioma, and although he sees daily a considerable number of cases of uterine disease he has never yet come across a case of malignant adenoma or of scirrhus affecting this organ. Yet it is recorded in a recent number of the *British Medical Journal* that one operator pronounced two out of three cases to be "scirrhus." Dr. Bell looks upon these as being instances of error in diagnosis; the probability is that they consisted of fibromatous interstitial nodules, which gave rise to the symptoms suggestive of cancer. If scirrhus was ever met with in the uterus he would conclude that it had arisen from metastasis, and would therefore not be amenable to operation. He could quite as readily conceive of scirrhus appearing as a primary disease on the tongue, lips or rectum.

The natural history of epithelioma, he says, differs materially from that of scirrhus; the former invariably attacks a tissue which has been debilitated by pre-existing disease, and in its structure is closely allied to the tissue from which it originates; scirrhus, on the other hand, manifests itself in the majority of instances in tissues where there has been an entire absence of indications which would lead one to prognosticate its advent. Moreover, there are sufficient grounds for supposing that scirrhus is the result of the transmigration of a cell or cells foreign to the part which is attacked. Epithelioma, therefore, would seem to be the result, in the first instance, of an aberrated condition of the matrix of the epithelium, the consequence being that abnormal instead of normal cells are generated, and their proliferation is increased pro rata. The same symptoms are manifested in myxedema, which is invariably accompanied by metrorrhagia to a greater or lesser extent. When this disease is treated by means of thyroid gland the epithelium is restored to health, and as a consequence the hemorrhage ceases.

The relation which exist between the thyroid body and the genital organs (if one is to credit Catulle's statement), says Dr. Bell, was well known to the ancients. It has also been demonstrated that pregnancy, when accompanied by hypertrophy of the thyroid body, sometimes favors the regression of a pre-existing thyroid tumor; Professor Charcot, having seen women affected with exophthalmic goitre cured after pregnancy, did not fail to advise it, on therapeutic grounds, in the treatment of this painful affection. (Dr. Bell's experience, however, is that women suffering from goitre have been less prone to become pregnant than other women.) The relationship between the thyroid body and the uterus is affirmed in a more decisive manner by certain singular facts observed by Bouilly, Tuffier, Guinard, Picque and Bloch, these surgeons having had to operate on patients affected by fibroma of the uterus or salpingo-ophoritis in whom co-existed goitres, some of which had previously resisted all treatment, have seen thyroid tumors disappear, or at least be considerably diminished, after extirpation of the pelvic organs.

If, now, continues Dr. Bell, we study the etiology of cancer of the uterus, for example, we find among the predisposing causes all those which can contribute to depress the vitality of the organ. He refers especially to repeated attacks of metritis, but more especially to endometritis resulting from other idiopathic or traumatic causes. If we bear in mind, therefore, the pathological relationship of the thyroid body to the womb, we may readily admit, he says, that the vitality of the latter may depend to a large extent on the integrity of the thyroid function. One is much more inclined to favor this opinion when one studies the results which many authors have obtained who have had a large experience in the treatment of myxedema, and who testify to the fact just stated that their cases have been frequently accompanied by metrorrhagia. It is these considerations which prompted Dr. Bell in the first instance to treat cancer of the uterus by the ingestion of fresh thyroid gland. It must be understood, however, that the local treatment mentioned in the beginning of this article is not to be neglected. He does not mention that this method of treatment is a panacea; what he maintains is that it is a remedy which deserves to be placed on record, and which will prove quite as reliable as the majority of remedies for other diseases.

When the disease has been in its initial stage the results have been more than satisfactory, and in several instances, even where considerable inroads have been made and where a cure could hardly be expected, the parts have returned to their normal condition.

His argument is that where surgical interference can be of avail this therapeutic method of t eatment is almost certain of success, so that the ultimate condition of the patient is very much more satisfactory than after a hysterectomy, while all the risks and the anxiety of the operation have been avoided.

The treatment of ovarian and tubal disease, as also that of fibroma of the uterus, by means of parotid and mammary gland has been suggested, he says, by physiological relationship which exists between these organs.

The effect of the parotid gland upon a diseased ovary is so pronounced, especially if the uterine affection is simultaneously treated, that it would be wrong, in his opinion, not to employ this remedy before resorting to operation. During the past two years he has obtained most favorable results in over sixty cases of enlarged and painful ovaries which would certainly at one time have warranted oophorectomy. These women are at the present moment not only perfectly well and free from pain, but, what is equally satisfatory, they remain also in possession of their ovaries.—*N. A. Practitioner.*

